# Factors Affecting Preventive Dental Treatment of Adolescents in Korea

**DOI:** 10.3390/ijerph17144948

**Published:** 2020-07-09

**Authors:** Seon-Hui Kwak, Soo-Myoung Bae, Sun-Jung Shin, Bo-Mi Shin

**Affiliations:** 1 Department of Dental Hygiene, College of Dentistry, Gangneung Wonju National University, Gangneung-si 25457, Korea; tjsgml0617@hanmail.net (S.-H.K.); edelweiss@gwnu.ac.kr (S.-M.B.); freshjung@gwnu.ac.kr (S.-J.S.); 2 Research Institute of Oral Science, Gangneung Wonju National University, Gangneung-si 25457, Korea

**Keywords:** adolescent health, multilevel analysis, oral health, social context, social health determinants

## Abstract

We conducted a multilevel analysis to identify factors affecting adolescents’ preventive dental treatment experience in South Korea. We sampled 72,435 students who participated in the 9th Korea Youth Risk Behavior Web-based Survey. The individual-level variables were divided into demographic factors, socioeconomic factors, oral health behavioral factors, and oral symptom experience factors. The regional-level variables included oral health resources, rate of students receiving oral health education at school by region, social deprivation index, and the number of private educational institutions. A higher rate of receiving oral health education in school by region was associated with increased fluoride application (1.04 times, *p* = 0.003). However, the number of private educational institutes per 1000 people was negatively associated with fluoride application experienced by students (0.64 times, *p* = 0.039). Students underwent more scaling when there were more dentists per 10,000 individuals (1.14 times, *p* = 0.008) and less scaling in areas with a higher social deprivation index (0.88 times, *p* = 0.024). To increase the access to preventive care for oral diseases among adolescents, a preventive system should be established in schools, and a primary dental care system should be established through the cooperation of the government, private dental clinics, and schools.

## 1. Introduction

Adolescence is the period during which the differentiation of permanent teeth, which must be used lifelong, is completed and is the most appropriate time to prevent oral diseases through correct oral health behaviors and preventive treatment. It is also a period with high risk for oral diseases, depending on individual factors such as the socioeconomic level of the family and eating habits, as well as environmental factors in schools and regions of residence [1,2,3]. Therefore, it is essential to develop preventive interventions in relation to social health determinants in children and adolescents in order to form correct oral health behaviors and promote oral health throughout life [4,5].

Individuals can form health behaviors influenced by their social relationships with people in their groups and the surrounding environment of the region. For example, in areas where social relations are systematically established and where social resources are abundant, local residents work together to improve their physical activities and create local spaces to reduce stress [6,7]. However, if public transportation is limited in poor areas and there are cigarette outlets in walking distance, people’s smoking cessation practices can be negatively affected [8,9].

Adults living in regions with easily accessible public health services and medical facilities are less likely to have dental caries. Lee et al. showed that the lower the community welfare budget and the number of dental clinics, the significantly higher the risk of dental caries [10]. Turrell and colleagues argued that the lack of public transportation could act as a barrier to the use of medical care in these regions, suggesting that policies and interventions that consider neighborhood-level factors are needed to increase access to medical care [11].

Regional-level factors are significant health determinants for adults, children, and adolescents. According to studies that identified factors affecting dental care utilization by children enrolled in US Medicaid [12], caregivers in poor socio-economic areas that lacked oral specialists were less likely to have access to dental care for their children, because of the low priority given to preventive care, despite the free dental benefits. Additionally, Fisher-Owens et al. showed that regional-level factors were associated with access to preventive dental care and dental health status among children; they further suggested that implementation of state policies, including care provided by dental health professionals, public dental health coverage, and public dental health programs, could reduce the differences across states [13].

Korea implemented the National Health Insurance Service (NHIS) for all citizens to increase access to medical services and to narrow the gap in medical utilization based on socioeconomic standards. Korea’s national health insurance covers all citizens living in Korea. The insured person pays the National Health Insurance Service monthly premiums according to their level of wealth, and all citizens are entitled to the same insurance coverage. If the insured person has received health service from a medical institution, the person must pay a copayment determined according to criteria, such as the type of medical institution, the type of service, the age of the patient, and so on (minimum of 5% up to 60%). Low-income citizens who have difficulty in making a living can avail themselves of the services through public assistance. Depending on their income, citizens who are eligible for public assistance can receive the medical services free of charge; however, they may pay up to 15% as copayment for some services. NHIS (National Health Insurance Service) pays the rest of the costs, excluding copayments.

In the field of dentistry, the national health insurance coverage applies only to certain categories of prosthetic, conservative, and preventive treatments. Preventive treatment includes scaling for adults aged ≥19 years and sealants for children and adolescents aged ≤18 years. Fluoride application and scaling for children and adolescents are not covered by the NHIS. Adults aged ≥19 years pay a 30% copayment for scaling, whereas children and adolescents aged ≤18 years with general insurance and those receiving public assistance pay 10% and 5%, respectively, for sealants [14].

In addition, the government provides preventive services to local residents for free through oral health programs in public health centers. The public health center provides school oral health education, fluoride solution brushing, and children’s fluoride application (gel or varnish) to prevent oral diseases in teenagers and improve oral health practices. In addition, socioeconomically vulnerable areas have established school dental clinics to provide elementary students with comprehensive preventive care, including oral examinations, sealants, oral health education, and simple preservation treatment [15]. However, most of these national interventions are conducted for primary school students, and the participation of secondary schools is insufficient. As a result, the number of decayed, missing, and filled teeth among Korean 15-year-olds was 2.33, higher than that in developed countries, such as the USA (1.2), the UK (0.8), and Denmark (0.4) [16,17].

Oral disease can be prevented through appropriate preventive interventions for related factors; thus, there is a need for concerted efforts from individuals, communities, and governments [18]. Effective intervention requires a multilevel approach considering the social, cultural, and institutional characteristics of groups as well as individuals [19]. Although many previous studies have investigated the influence of regional-level factors on child oral health status [11,20], oral health-related quality of life of children [21], and adult oral health behavior [22], few studies have identified regional-level factors affecting preventive dental treatment in Korean adolescents in Asia. Therefore, this study aimed to investigate whether there are any regional factors affecting Korean adolescents’ preventive dental treatment experience by performing a multi-level analysis using national statistical data representing all Korean adolescents.

## 2. Materials and Methods

### 2.1. Study Population

This study analyzed secondary data using the results of an anonymous self-report questionnaire of 72,435 students (13- to 18-year-olds) from 800 middle and high schools nationwide participating in the 9th Korea Youth Risk Behavior Web-based Survey (KYRBWS) in 2013. The KYRBWS was a government-approved statistical survey conducted for students from 1st grade in middle school to 3rd grade in high school to understand the health behaviors, including oral health, smoking, and drinking, of Korean adolescents in 15 areas.

The survey was designed with stratified multistage cluster-sampling that considered regions and schools to minimize sample errors. The 800 middle and high schools from 16 cities nationwide were selected by applying the proportional distribution method [23]. Students currently enrolled in the 800 selected sample schools accessed the website with the participation number given to each of them, read the survey participation agreement that appeared on the screen, and clicked the participate button to take a survey, if they agreed to participate in the survey. The survey was conducted from 1 June to 30 July, 2013. Since 2005, the Ministry of Education, the Ministry of Health and Welfare, and the Centers for Disease Control in Korea have conducted an annual government-approved statistical survey. The KYRBWS was approved by the Institutional Bioethics Committee of the Korea Centers for Disease Control and Prevention.

### 2.2. Dependent Variables

The annual professional fluoride application experience and the annual scaling experience were evaluated using two questions: “Have you received professional fluoride application in the last 12 months?” and “Have you undergone scaling in the last 12 months?” The annual fluoridation experience and scaling experience were defined when each question was answered “Yes.” The response “No” was excluded.

### 2.3. Individual-Level Variables

Individual-level independent variables, including demographic, socioeconomic, oral health behavioral, and oral symptom experience factors, were selected from the KYRBWS. The demographic variables used were gender, school type, school year, and grade. The school year was used to reflect the age of the student; the 1st year in middle school corresponds to 13 years of age, and the 3rd year in high school corresponds to 18 years of age.

Socioeconomic variables used were the father’s education level, family affluence scale (FAS), and weekly allowance. The father’s education level represented the parents’ education level. The FAS evaluated four questions and their response categories, and the questions and answers were as follows: “Does your family own a car?” (0 = no; 1 = yes; 2 = yes, 2 or more); “Do you have your own bedroom?” (0 = no; 1 = yes); “How many times did you travel away on holidays during the past year?” (0 = not at all; 1 = once; 2 = twice; 3 = more than twice); “How many computers does your family own?” (0 = none; 1 = one; 2 = two; 3 = more than 2).

Oral health behavior variables included snack consumption, brushing after lunch, and perceived oral health. Oral symptoms were assessed by asking, “Have you experienced tooth failure, toothache, gum disease, soft tissue disease, or bad breath during the past year?” (0 = no; 1 = yes). The oral symptom experience variable was the sum of experiences for each symptom. The summed values (range between 0 and 5) were converted to 0 and 1 (0 = no; 1 = one or more). The answer “not sure” was treated as a missing value [23,24].

### 2.4. Regional-Level Variables

Regional-level variables were constructed using National Statistical Data to represent the socio-economic characteristics of 16 regions, dental medical resources, preventive service beneficiaries, and the living environment of adolescents. In this study, because the KYRBWS used the status of 16 cities/provinces as individual-level variables, regional-level variables were made to match the 16 cities/provinces.

The socio-economic level of a region is determined using the social deprivation index. The social deprivation index is a composite index calculated from indicators such as residential density, housing ownership, car ownership, regional income, education level, residential facility type, and the residential environment as the average z-score provided in the population census data. This can indicate not only the lack of local financial resources but also the deficiencies of living conditions and facilities [25].

The number of dental clinics/hospitals per 10,000 population and the number of dentists per 10,000 population were used for assessing dental resources. The data used included the National Statistical Data and the statistics provided by the Korean Dental Association [26,27]. Dental resources are an indicator that can confirm access to medical use by local residents [28].

The rate of students receiving oral health education at schools by region was used to identify local priorities for oral health in adolescents. School oral health education is managed by the public health center; therefore, the higher the school oral health education beneficiary rate, the higher the interest in youth’s oral health. These data were assessed by reorganizing the proportion of students who answered “Yes” for the question “Have you had oral health education at school for the past year?” in the KYRBWS data by region [23].

Private educational institutes are institutions that teach math, English, and science outside the school, and Korean middle and high school students spend time studying at private institutes after school or on weekends. Therefore, the number of private educational institutes per 1000 population was used as an index reflecting the lifestyle of Korean adolescents living in a social environment focused on the college entrance examination [26,28].

### 2.5. Statistical Analysis

A two-level analysis was conducted to identify factors at individual and regional levels that influenced the preventive dental treatment experience of Korean adolescents. In this study, since the dependent variable had a Bernoulli distribution as a binomial variable, multilevel logistic regression analysis was performed to convert probability to logit values, which were expressed as a linear relationship. For the independent variable, the individual-level factor variable was used as the 1-level unit, and the regional-level factor variable was used as the 2-level unit.

In terms of statistical analysis, the professional fluoride application and scaling experiences were set as dependent variables and were analyzed by dividing them into three stages, such as the null model, unconditional slope model, and conditional model. Because the null model is a process of verifying that the dependent variable differs between regions, only the dependent variable was used, and the intraclass correlation coefficient (ICC) was confirmed. Since the dependent variable was a binary variable, ICC was calculated by the formula:

ICC = τ_00_ (2-level variance)/[τ_00_ (2-level variance) + (π^2^/3)] (1)

The unconditional slope model is a process of checking the random and fixed effects of individual-level variables and inputs individual-level independent variables with statistically significant associations between dependent variables and complex sample logistic regression. Finally, the conditional model is a process of verifying individual and regional factors affecting independent variables, and therefore, both individual- and regional-level independent variables are used. The model with the highest explanatory power for the dependent variable was finally selected and presented in the results. A likelihood ratio test was performed to test the model’s suitability. Using the maximum likelihood method, -2LL (log likelihood) was used to check the deviance; the smaller the deviance value, the better the model’s suitability and validity. Statistical analysis was performed using SPSS 23.0 (SPSS Inc., Chicago, IL, USA) and HLM 7.0 (Scientific Software International Inc., Chicago, IL, USA) programs.

This study obtained approval from the Institutional Bioethics Committee of the Korea Centers for Disease Control and Prevention for the use of non-identifiable secondary data and from the Bioethics Committee of Gangneung-Wonju National University (IRB No. GWNUIRB-2016-09).

## 3. Results

### 3.1. Annual Professional Fluoride Application and Scaling Experience Rate

Professional fluoride application was more often experienced by younger adolescents, and scaling was more frequent in older adolescents (*p* < 0.001). In addition, the higher the grade, the more preventive treatment was experienced (*p* < 0.001). Regarding socio-economic factors, the higher the father’s education level, and the higher the FAS level, the higher the experience rate (*p* < 0.05). Regarding oral health behavioral factors, students who used to brush after lunch always/almost always, when eating snacks more than three times a week, experienced more fluoride application and scaling. In addition, when students experienced oral health symptoms such as tooth fracture, toothache, gingival bleeding, soft tissue pain, and bad breath, the rate of preventive treatment was high (Table 1).

### 3.2. Relationship Between Personal Characteristics, Annual Professional Fluoride Application, and Scaling Experience

After adjusting for demographic factors, high-FAS-level students experienced 1.50 times more fluoride applications than those with low FAS levels (*p* < 0.001), and students that received an allowance of more than 20,000 won per week experienced 1.11 times more fluoride applications than those who received less than 10,000 won per week (*p* = 0.002). Regarding oral health behavior factors, the more frequent the tooth brushing after lunch, the more fluoride application was experienced (<0.001) (Table 2).

After adjusting for demographic factors, students with a higher paternal education level experienced 1.28 times more scaling. In addition, students with high FAS levels were found to experience 1.90 times more scaling than those with low FAS levels (*p* < 0.001). Regarding oral health behavior factors, increased tooth-brushing after lunch was associated with more scaling (*p* < 0.001).

### 3.3. Multilevel Analysis of Annual Professional Fluoride Application Experience

The deviance of the final model was 178,160.92, which was lower than that of the null model (198,038.68). Regional-level factors affecting annual professional fluoride application were found to be the rate of receiving oral health education at school by region and the number of private educational institutes per 1000 people. A higher rate of students receiving oral health education at school by region was associated with increased fluoride applications (1.04 times, *p* = 0.003).

On the other hand, more private educational institutes per 1000 people were associated with fewer fluoride applications (0.64 times, *p* = 0.039). Regarding the annual experience of professional fluoride application, the annual experience of adolescents with gum bleeding was 1.19 times more than that of adolescents without gum bleeding (*p* < 0.001) (Table 3).

### 3.4. Multilevel Analysis of Annual Scaling Experience

The deviance of the final model was 165,244.34, which was decreased compared to that of the null model (207,474.60). The regional-level factors affecting the annual scaling experience were the number of dentists per 10,000 individuals and the social deprivation index. The experience of scaling treatments was higher when there were more dentists per 10,000 individuals (1.14 times, *p* = 0.008), while a higher social deprivation index was linked to fewer scaling treatments (0.88 times, *p* = 0.024). The annual scaling experience was found to be 1.29 times higher for adolescents with gum bleeding than for those without gum bleeding (*p* < 0.001) (Table 4).

## 4. Discussion

This study identified the individual- and regional-level factors that influence preventive dental treatment in Korean adolescents by using government-approved National Statistical Data that is representative of Korean adolescents.

The annual professional fluoride application experience was found to be 1.04 times higher in areas with a higher rate of receiving school oral health education by region. These findings were consistent with previous studies that showed that enhancing children’s access to medical care in community-based programs was associated with increased preventive care [29].

In Korea, school health education is conducted in two ways. First, as part of the oral health program under the Oral Health Act of Korea, dental hygienists working at public health centers visit elementary, middle, and high schools to provide oral health education to students in schools [30]. The health promotion activities of a community may vary depending on the policies of the local government and the characteristics of local residents [31]. If there is a problem with the health condition and behavior of the local adolescents or if the residents’ demand for youth health is high, the health center will be interested in the students’ health and will implement the health programs. Second, through a ‘health’ class, which is one of the school curricula, a school nurse provides oral health education [32]. The middle and high schools in Korea consider a university entrance-oriented education as the top priority and conduct classes according to this orientation. The Ministry of Education in Korea sets the health class as an elective or liberal arts class, and allows it to be conducted at the discretion of the principal [32]. In fact, high school students only do self-study for subjects not included in university entrance exams, which focus on subjects such as Korean, math, English, science, and social science.

Despite this social environment, the high proportion of oral health education observed in local schools means that principals who oversee schools’ class curriculum are interested in and consider students’ oral health important. The high percentage of individuals who have benefited from local schools’ oral health education means that local schools and health public centers have a high interest in adolescents’ oral health. Therefore, these results may indicate that public health centers’ and schools’ interest in oral health of the students can significantly affect adolescents’ preventive treatment experience.

In this study, adolescents living in regions with more private educational institutes experienced 36% fewer professional fluoride applications. The results of this study suggest that the greater the commitment to learning in a region, the lower the interest in and the priority given to preventive care, and the less likely that such care is to be experienced. In a previous study, which was analyzed using the Korean National Health and Nutrition Survey data, the main cause of the unmet dental care needs in adolescents was that “the school could not be vacated (male and female average 35.94%);” this finding supports the results of this study [33]. In Korea, adolescents spend more than half of their daily life studying, which implies that their physical and mental health is poor. According to the Organization for Economic Co-operation and Development (OECD)’s Program for International Student Assessment, 23% of Korean adolescents spend at least 60 h per week studying, both in and out of school (OECD average: 13%) [34]. Moreover, after school, students are engaged in an additional 2–5 h of extra-curricular activities. Because of this academic-oriented lifestyle, Korean adolescents have high levels of stress with regard to poor grades, with a low percentage of leisure time and physical activity [35]. Additionally, higher academic stress leads to increased unhealthy behaviors, such as smoking and drinking [36].

The World Health Organization (WHO) encourages schools to promote health by creating a physical environment in which the health risks of students can be addressed, including regular preventive dental care [37]. In 2009, Korea started a health promotion school project, and as of 2013, it was operative in 85 schools [38]. This accounts for only 0.7% of the total number of schools, and as it mainly focuses on general health, oral health intervention has been insufficient. In order to solve the problems of academic stress, unhealthy behavior, and unmet dental care among the youth, it is necessary to activate the WHO-recommended health promotion school program.

Adolescents in regions with more dentists per 10,000 individuals were found to experience 1.14 times more scaling. Additionally, with a higher social deprivation index, 12% fewer scaling treatments were experienced. This was consistent with previous studies, which reported that regions with lower socioeconomic levels had less access to medical resources because dental resources were concentrated in large metropolitan regions with high socioeconomic standards [13]. The number of dentists in the region was significantly associated with the annual scaling experience, but not with the annual professional fluoride application experience.

In 2013, regarding the ranking of the total cost of medical care benefits by disease among adolescents aged 15 to 19 years, dental caries was reported to rank fourth, whereas gingivitis and periodontal disease ranked sixth [39]. This report means that many adolescents visited the dental clinic when they had oral symptoms, such as dental caries and gingivitis, and paid for and received treatment from the dentist. In addition, Korea’s national health insurance mainly covers treatment items. Preventive measures, such as professional fluoride application, are not covered by the national health insurance; thus, dental visits for treatments are more frequent. Therefore, the number of dentists only affects the scaling experience, which is considered to be the effect of Korea’s treatment-oriented medical system. It is also thought that the impact of the number of dentists in the region on preventive care is small and contributes to improving access to dental visits for treatment.

The WHO encourages schools to run disease prevention and health promotion programs because schools are well placed to promote lifelong healthy behaviors and serve as a platform to access families and communities [40]. In the USA, school-based health centers offer comprehensive services to students and families at minimal or no cost [41]. In Georgia, USA, legislation has been enacted to allow dental hygienists to provide preventive care in schools if there are few dentists in an area and the distance from dental services is significant [42]. In Korea, the School Oral Health Center is currently installed in only 2.8% of 6145 elementary and special schools nationwide, centered on vulnerable socio-economic areas, and middle and high schools do not have this facility [43]. There are no full-time dentists and dental hygienists in school dental clinics, and dentists and dental hygienists working at the local public health center regularly go on business trips to schools, providing students with preventive care such as oral examinations, fluoride applications (solution, gel, or varnish), sealants, scaling, and education. As of 2019, 1130 dental hygienists and 420 dentists are employed in South Korea’s public health sector [26]. If school dental clinics are expanded and operated in the same way as they are now, it is expected that there will be a shortage of dental workforce. Therefore, the government should support the expansion of oral health programs by seeking ways to utilize the dental workforce in the public sector with related associations.

In order to promote the oral health of middle and high school students, active participation and changes in public health centers, associations, and government are necessary. To promote school-based oral health, the related government agencies, such as the Ministry of Education and the Ministry of Health and Welfare, should promote cooperation and joint research with specialized institutions, such as the Dentist Association and the Dental Hygiene Association, to develop standardized school-based education programs. Furthermore, the developed programs should be piloted, and the results should be assessed to accumulate evidence. In addition, they should also work on legal measures to enable dentists or dental hygienists to provide comprehensive oral care services to students full time in schools, similar to the practice in developed countries. Public health center officials should be interested in the oral health of adolescents and implement oral health promotion programs for adolescents.

This study was limited in that several factors could not be considered, given that it utilized secondary data, and actual dental treatment data were not used. In addition, this study used self-completed surveys on dental treatment experience, which could be confounded by re-call and subjective interpretations. To identify the factors related to adolescents’ oral health behavior, it is necessary to conduct a multilevel analysis that encompasses three levels (individual, school, and region levels) by adding school-level factors, as teenagers spend most of their time in school. Nevertheless, this study was meaningful in that it identified factors that affect the preventive treatment experience of Korean adolescents at individual and regional levels by using National Statistics Data that are representative of Korean adolescents.

## 5. Conclusions

This study confirmed that adolescents’ access to preventive dental treatment was influenced not only by individual level factors but also by regional factors. To improve access to preventive care for Korean adolescents, it is necessary to establish a primary dental care system through the cooperation of government, private dental clinics, and schools, so that students can regularly receive dental care. In socioeconomically vulnerable regions with low access to dental care, dental teams should be utilized through the school environment to create a system that provides students with continuous oral health care, and to establish a medical delivery system where health centers and private dentists can collaborate to provide treatment if necessary.

## Figures and Tables

**Table 1:** Annual professional fluoride application and scaling experience rate according to general characteristics.

Variable			Total	Professional Fluoride Application		Scaling	
			N (wt %)	N (wt %)	*p*-Value	N (wt %)	*p*-Value
Total			72,435 (100.0)	10,036 (12.3)		15,250 (21.5)	
Demographic factors	Sex	Boy	36,655 (52.3)	4924 (11.8)	0.113	7085 (19.8)	<0.001
		Girl	35,780 (47.7)	5112 (12.9)		8165 (23.4)	
	School type	Boys’ school	12,512 (17.6)	1626 (13.1)	0.475	2662 (21.3)	0.367
		Girls’ school	12,695 (17.0)	1812 (12.9)		2670 (21.0)	
		Co-ed school	47,228 (65.4)	6598 (11.9)		9918 (21.7)	
	School year	3rd in high school	12,012 (17.2)	973 (7.2)	<0.001	2666 (22.6)	<0.001
		2nd in high school	11,865 (16.9)	1173 (9.0)		2761 (23.6)	
		1st in high school	12,028 (17.1)	1324 (9.9)		2574 (21.6)	
		3rd in middle school	12,218 (16.5)	1733 (12.2)		2462 (20.8)	
		2nd in middle school	12,133 (16.0)	2068 (15.5)		2516 (21.3)	
		1st in middle school	12,199 (16.2)	2765 (20.7)		2271 (19.1)	
	Grade	Low	27,292 (37.3)	3423 (11.0)	<0.001	5142 (19.2)	<0.001
		Middle	20,148 (28.1)	2751 (12.1)		4161 (21.0)	
		High	24,995 (34.6)	3862 (13.9)		5947 (24.4)	
Socio-economic factors	Fathers’ education level	Less than high school graduate	26,686 (43.5)	3667 (11.6)	0.010	5070 (19.3)	<0.001
		More than college graduate	31,362 (26.5)	4242 (12.6)		7755 (25.0)	
	Family affluence scale	Low	8541 (11.3)	973 (9.4)	<0.001	1210 (14.6)	<0.001
		Middle	37,767 (51.7)	4749 (10.9)		7300 (19.8)	
		High	26,127 (37.0)	4314 (15.2)		6740 (26.1)	
	Weekly allowance	<10,000	23,218 (32.0)	3443 (13.1)	<0.001	4625 (20.5)	<0.001
		10,000–19,999	18,995 (26.0)	2637 (12.3)		3796 (20.5)	
		≥20,000	30,222 (42.0)	3956 (11.7)		6829 (22.9)	
Oral health behavior factors	Snack consumption	Less twice a week	43,999 (61.2)	5792 (11.6)	<0.001	8891 (20.7)	<0.001
		3 or more times a week	28,436 (38.8)	4244 (13.5)		6359 (22.8)	
	Tooth brushing after lunch	Sometimes/No	29,893 (43.6)	3564 (11.0)	<0.001	5751 (19.7)	<0.001
		Always/Almost always	42,542 (56.4)	6472 (13.3)		9499 (22.9)	
	Perceived oral health status	Healthy	57,312 (79.3)	7985 (12.4)	0.347	11,807 (21.1)	<0.001
		Poor	15,123 (20.7)	2051 (12.1)		3443 (23.1)	
	Oral symptoms experience	No	27,322 (37.9)	3622 (11.7)	<0.001	4686 (17.6)	<0.001
		1 or more	45,113 (62.1)	6414 (12.7)		10,564 (23.9)	
	Troubled by tooth fracture	No	63,253 (87.6)	8644 (12.2)	0.007	13,032 (21.1)	<0.001
		Yes	9182 (12.4)	1392 (13.2)		2218 (24.5)	
	Toothache while eating foods	No	44,884 (62.0)	6062 (11.9)	<0.001	8538 (19.5)	<0.001
		Yes	27,551 (38.0)	3974 (12.9)		6712 (24.8)	
	Teeth ache all the time	No	52,532 (72.6)	7166 (12.1)	0.006	10,162 (19.8)	<0.001
		Yes	19,903 (27.4)	2870 (12.9)		5086 (26.0)	
	Bleeding gum	No	58,173 (80.5)	7867 (12.0)	<0.001	11,432 (20.1)	<0.001
		Yes	14,262 (19.5)	2169 (13.7)		3818 (27.4)	
	Aching inside of the mouth or tongue	No	63,964 (88.2)	8669 (12.0)	<0.001	12,899 (20.6)	<0.001
		Yes	8471 (11.8)	1367 (14.8)		2351 (28.3)	
	Bad breath	No	56,185 (77.6)	7692 (12.1)	0.004	11,337 (20.6)	<0.001
		Yes	16,250 (22.4)	2344 (13.0)		3913 (24.7)	

The data were analyzed by complex sample analysis (chi-squared test).

**Table 2:** Relationship between personal characteristics and annual profession fluoride application and scaling experience.

Variable			Professional Fluoride Application		Scaling	
			OR (95% CI)	*p*-Value	OR (95% CI)	*p*-Value
Demographic factors	Sex	Boy	–	–	Ref	
		Girl	–	–	1.15 (1.09–1.21)	<0.001
	School type	Boys’ school	–	–	–	
		Girls’ school	–	–	–	–
		Co–ed school	–	–	–	–
	School year	3rd in high school	Ref		Ref	
		2nd in high school	1.34 (1.20–1.48)	<0.001	1.08 (1.01–1.16)	0.022
		1st in high school	1.57 (1.34–1.83)	<0.001	0.97 (0.91–1.04)	0.420
		3rd in middle school	2.06 (1.68–2.53)	<0.001	0.90 (0.84–0.97)	0.007
		2nd in middle school	2.86 (2.36–3.47)	<0.001	0.96 (0.89–1.04)	0.320
		1st in middle school	4.00 (3.33–4.81)	<0.001	0.85 (0.78–0.92)	<0.001
	Grade	Low	Ref		Ref	
		Middle	1.08 (1.00–1.15)	<0.001	1.08 (1.03–1.14)	0.004
		High	1.18 (1.10–1.26)	0.040	1.24 (1.18–1.30)	<0.001
Socio–economic factors	Fathers’ education level	Less than high school graduate	Ref		Ref	
		More than college graduate	0.94 (0.88–1.01)	0.080	1.28 (1.22–1.34)	<0.001
	Family affluence scale	Low	Ref		Ref	
		Middle	1.18 (1.07–1.31)	0.002	1.37 (1.27–1.48)	<0.001
		High	1.50 (1.35–1.67)	<0.001	1.90 (1.75–2.06)	<0.001
	Weekly allowance (won)	<10,000	Ref		Ref	
		10,000–19,999	1.08 (1.01–1.16)	0.026	0.98 (0.93–1.03)	0.429
		≥20,000	1.11 (1.04–1.19)	0.002	1.06 (1.01–1.11)	0.025
Oral health behavior factors	Snack consumption	Less twice a week	Ref		Ref	
		3 or more times a week	1.13 (1.07–1.20)	<0.001	1.06 (1.02–1.11)	0.006
	Tooth brushing after lunch	Sometimes/No	Ref		Ref	
		Always/Almost always	1.49 (1.34–1.66)	<0.001	1.11 (1.06–1.16)	<0.001
	Perceived oral health status	Healthy	–	–	Ref	
		Poor	–	–	1.01 (0.95–1.06)	0.812
	Oral symptoms experience	No	Ref		Ref	
		1 or more	1.06 (0.97–1.16)	0.177	1.13 (1.06–1.21)	<0.001
	Troubled by tooth fracture	No	Ref		Ref	
		Yes	1.04 (0.96–1.13)	0.379	1.15 (1.08–1.22)	<0.001
	Toothache while eating foods	No	Ref		Ref	
		Yes	1.04 (0.97–1.12)	0.280	1.09 (1.04–1.15)	0.001
	Teeth ache all the time	No	Ref		Ref	
		Yes	1.04 (0.98–1.11)	0.226	1.10 (1.05–1.16)	<0.001
	Bleeding gum	No	Ref		Ref	
		Yes	1.15 (1.07–1.22)	<0.001	1.25 (1.19–1.32)	<0.001
	Aching inside of the mouth or tongue	No	Ref		Ref	
		Yes	1.13 (1.05–1.22)	0.002	1.14 (1.07–1.21)	<0.001
	Bad breath	No	Ref		Ref	
		Yes	1.08 (1.01–1.15)	0.019	1.09 (1.04–1.15)	0.001

The data were analyzed by complex sample logistic regression. Values are presented as odds ratio (95% confidence interval). OR, odds ratio; CI, confidence interval.

**Table 3:** Multilevel analysis of annual professional fluoride application experience considering individual and regional characteristics.

Model Parameter		Model 1 Null Model		Model 2 Unconditional Slope Model		Model 3 Final Model	
		Odds Ratio (95% CI)	*p*-Value	Odds Ratio (95% CI)	*p*-Value	Odds Ratio (95% CI)	*p*-Value
Fixed effect							
level 1	Intercept *γ*_00_	0.18 (0.13–0.25)	<0.001	0.09 (0.06–0.13)	<0.001	0.07 (0.05–0.09)	<0.001
	Sex (girl vs. boy)			1.01 (0.95–1.08)	0.712	1.02 (0.98–1.06)	0.293
	School type (girls’ school vs. boys’ school)			–	–	–	–
	School type (co-ed school vs. boys’ school)			–	–	–	–
	School year (2nd in high school vs. 3rd in high school)			1.20 (1.06–1.37)	0.008	1.19 (1.04–1.37)	0.016
	School year (1st in high school vs. 3rd in high school)			1.46 (1.23–1.73)	<0.001	1.45 (1.22–1.72)	<0.001
	School year (3rd in middle school vs. 3rd in high school)			2.08 (1.52–2.86)	<0.001	2.29 (1.66–3.15)	<0.001
	School year (2nd in middle school vs. 3rd in high school)			2.39 (1.75–3.24)	<0.001	2.69 (1.97–3.68)	<0.001
	School year (1st in middle school vs. 3rd in high school)			3.10 (2.23–4.31)	<0.001	3.71 (2.67–5.17)	<0.001
	Grade (middle vs. low)			1.08 (1.00–1.17)	0.049	1.09 (1.01–1.18)	0.036
	Grade (high vs. low)			1.17 (1.07–1.28)	0.002	1.21 (1.11–1.33)	<0.001
	Fathers’ education level (More than college graduates vs. Less than high school graduate)			–	–	–	–
	Family affluence scale (middle vs. low)			0.95 (0.84–1.07)	0.382	0.97 (0.86–1.09)	0.588
	Family affluence scale (high vs. low)			1.04 (0.88–1.22)	0.650	1.10 (0.93–1.29)	0.240
	Weekly allowance (10,000–19,999 vs. <10,000 won)			1.01 (0.96–1.07)	0.678	–	–
	Weekly allowance (≥20,000 vs. <10,000 won)			1.01 (0.95–1.08)	0.721	–	–
	Snack consumption (3 or more times a week vs. less than twice a week)			1.05 (0.97–1.13)	0.221	–	–
	Tooth brushing after lunch (always/almost always vs. sometimes/no)			1.32 (1.22–1.43)	<0.001	1.37 (1.26–1.49)	<0.001
	Perceived oral health status (poor vs. healthy)			–	–	–	–
	oral symptoms Experience (1 or more vs. no)			–	–	–	–
	Teeth ache all the time (yes vs. no)			–	–	–	–
	Bleeding gums (yes vs. no)			1.12 (1.05–1.19)	0.001	1.19 (1.14–1.24)	<0.001
level 2	Number of dental clinics per 10,000 population					0.91 (0.77–1.09)	0.268
	Rate of receiving school-based oral health education by region					1.04 (1.02–1.06)	0.003
	Deprivation index					1.04 (0.83–1.32)	0.699
	Number of private educational institutes per 1000 population					0.64 (0.42–0.97)	0.039
Random effect							
τ_00_ = var (u_0j_) intercept variance		0.38	<0.001	0.37	<0.001	0.22	<0.001
Intra–Class Correlation (ICC)		0.104		0.101		0.063	
Deviance		198,038.68		170,844.48		178,160.92	
Reliability		0.993		0.830		0.779	

The data were analyzed by multilevel logistic regression. Values are presented as odds ratio (95% confidence interval). OR, odds ratio; CI, confidence interval.

**Table 4:** Multilevel analysis of annual scaling experience considering individual and regional characteristics.

Model Parameter		Model 1 Null Model		Model 2 Unconditional Slope Model		Model 3 Final Model	
		Odds Ratio (95% CI)	*p*-Value	Odds Ratio (95% CI)	*p*-Value	Odds Ratio (95% CI)	*p*-Value
Fixed effect							
level 1	Intercept *γ_00_*	0.25 (0.23–0.28)	<0.001	0.09 (0.08–0.11)	<0.001	0.09 (0.08–0.10)	<0.001
	Sex (girl vs. boy)			1.11 (1.05–1.18)	0.001	1.13 (1.08–1.18)	<0.001
	School type (girls’ school vs. boys’ school)			–	–	–	–
	School type (co-ed school vs. boys’ school)			–	–	–	–
	School year (2nd in high school vs. 3rd in high school)			1.05 (0.97–1.15)	0.215	1.06 (0.99–1.13)	0.092
	School year (1st in high school vs. 3rd in high school)			0.98 (0.89–1.08)	0.713	0.98 (0.91–1.04)	0.481
	School year (3rd in middle school vs. 3rd in high school)			0.88 (0.80–0.98)	0.019	0.90 (0.84–0.96)	0.002
	School year (2nd in middle school vs. 3rd in high school)			0.96 (0.87–1.07)	0.459	0.97 (0.90–1.04)	0.399
	School year (1st in middle school vs. 3rd in high school)			0.86 (0.78–0.95)	0.006	0.85 (0.79–0.92)	<0.001
	Grade (middle vs. low)			1.05 (0.99–1.13)	0.118	1.07 (1.01–1.12)	0.017
	Grade (high vs. low)			1.19 (1.12–1.27)	<0.001	1.21 (1.15–1.27)	<0.001
	Fathers’ education level (More than college graduates vs. Less than high school graduate)			1.23 (1.16–1.31)	<0.001	1.24 (1.19–1.29)	<0.001
	Family affluence scale (middle vs. low)			1.38 (1.22–1.56)	<0.001	1.40 (1.29–1.52)	<0.001
	Family affluence scale (high vs. low)			1.94 (1.72–2.19)	<0.001	1.96 (1.80–2.13)	<0.001
	Weekly allowance (10,000–19,999 vs. <10,000 won)			0.99 (0.91–1.08)	0.831	0.98 (0.93–1.04)	0.544
	Weekly allowance (≥20,000 vs. <10,000 won)			1.07 (1.01–1.14)	0.028	1.07 (1.02–1.13)	0.004
	Snack consumption (3 or more times a week vs. less than twice a week)			1.08 (1.02–1.13)	0.009	1.09 (1.04–1.13)	<0.001
	Tooth brushing after lunch (always/almost always vs. sometimes/no)			1.18 (1.09–1.28)	<0.001	1.17 (1.08–1.26)	<0.001
	Perceived oral health status (poor vs. healthy)			–	–	–	–
	oral symptoms Experience (1 or more vs. no)			1.37 (1.28–1.46)	<0.001	1.34 (1.28–1.41)	<0.001
	Teeth ache all the time (yes vs. no)			–	–	–	–
	Bleeding gums (yes vs. no)			1.27 (1.19–1.36)	<0.001	1.29 (1.22–1.36)	<0.001
level 2	Number of dentists per 10,000 population					1.14 (1.04–1.25)	0.008
	Rate of receiving school-based oral health education by region					1.01 (1.00–1.02)	0.106
	Deprivation index					0.88 (0.80–0.98)	0.024
	Number of private educational institutes per 1000 population					1.02 (0.86–1.22)	0.788
Random effect							
τ_00_ = var (u_0j_) intercept variance		0.03	<0.01	0.02	0.233	0.004	0.022
Intra–Class Correlation (ICC)		0.009		0.006		0.001	
Deviance		207,474.60		160,982.88		165,244.34	
Reliability		0.935		0.276		0.371	

The data were analyzed by multilevel logistic regression. Values are presented as odds ratio (95% confidence interval). CI, confidence interval.
